# Supplementary material for: ESKD Risk Prediction Model in a Multicenter Chronic Kidney Disease Cohort in China: A Derivation, Validation, and Comparison Study
Source: J Clin Med. 2023 Feb 14;12(4):1504. doi: 10.3390/jcm12041504 (PMC9965616; doi:10.3390/jcm12041504)
Supplement: Supplementary file 1 [file jcm-12-01504-s001.zip › jcm-2157018-supplementary.pdf]

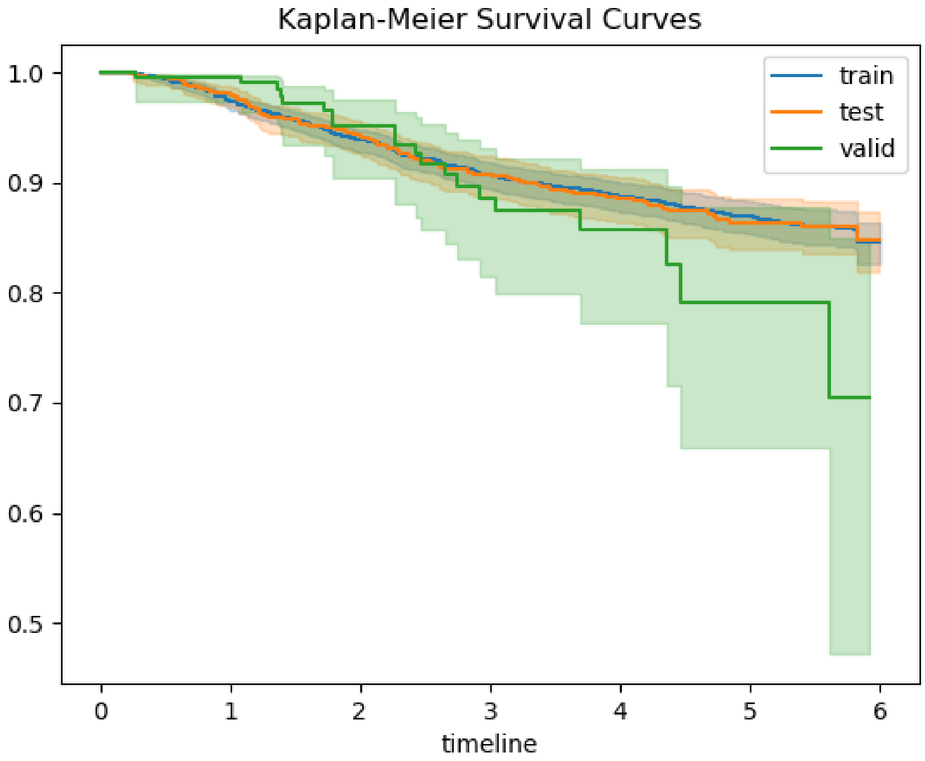


**Figure S1.** The Kaplan-Meier survival curves in training, testing, and validation dataset.

C-STRIDE was employed as the model's training (blue line) and testing (orange line) datasets with a split ratio of 7:3 (N=2251:965). PKUFH served as an independent external validation (green line) cohort (N=342).

**Table S1.** Multivariable Cox analysis using both directions step in the training dataset.

| Variables | coefficient | exp(coef) | se(coef) | *p*-value |
|---|---|---|---|---|
| Age, years | - 0.010 | 0.990 | 0.006 | 0.074 |
| Male | 0.394 | 1.483 | 0.167 | 0.038* |
| DBP, mmHg | 0.009 | 1.009 | 0.005 | 0.049* |
| HBP, n (%) | 0.860 | 2.364 | 0.261 | 0.001* |
| T2DM, n (%) | 0.405 | 1.500 | 0.147 | 0.024* |
| CVD, n (%) | 0.272 | 1.313 | 0.174 | 0.118 |
| ALB, g/L | - 0.028 | 1.902 | 0.008 | 0.001* |
| HGB, mg/L | - 0.012 | 0.988 | 0.003 | 0.000* |
| Creatinine, μmol/L | 0.005 | 1.005 | 0.002 | 0.006* |
| eGFR, mL/min/1.73 $m^2$ | - 0.015 | 0.985 | 0.007 | 0.026* |
| UACR_log, mg/g | 0.643 | 1.902 | 0.108 | 0.000* |
| HDL-C, mmol/L | - 0.413 | 0.662 | 0.206 | 0.123 |
| uCa, mmol/L | - 0.213 | 0.808 | 0.062 | 0.001* |

Abbreviations: coef, coefficient; HBP, hypertension; T2DM, type 2 diabetes mellitus; CVD: cardiovascular disease. ALB, albumin; HGB, hemoglobin; eGFR, estimated glomerular filtration rate; UACR, urinary albumin-creatinine ratio; HDL-C, high-density lipoprotein cholesterol; uCa: 24-hour urine total calcium.

**Table S2.** Feature selection in Cox-LASSO cross-validation with 1 standard error lambda.

| Variables | coefficient |
|---|---|
| Age, years | . |
| Male | . |
| DBP, mmHg | 0.004 |
| BMI Kg/m$^2$ | . |
| HBP, n (%) | 0.273 |
| T2DM, n (%) | 0.166 |
| CVD, n (%) | . |
| smoke, n (%) | . |
| ALB, g/L | - 0.023 |
| HGB, mg/L | - 0.009 |
| Serum Creatinine, μmol/L | 0.009 |
| eGFR, mL/min/1.73 m$^2$ | . |
| UACR_log, mg/g | 0.408 |
| HDL-C, mmol/L | . |
| uCa, mmol/L | - 0.213 |
| Uric acid, mmol/L | . |
| FBG, mmol/L | . |
| Triglyceride, mmol/L | . |
| TC, mmol/L | . |
| LDLC, mmol/L | . |
| Serum Calcium, mmol/L | . |
| Serum Potassium, mmol/L | . |
| Serum Phosphorus, mmol/L | . |

Abbreviations: DBP, diastolic blood pressure; BMI: body mass index; HBP, hypertension; T2DM, type 2 diabetes mellitus; CVD: cardiovascular disease. ALB, albumin; HGB, hemoglobin; eGFR, estimated glomerular filtration rate; UACR, urinary albumin-creatinine ratio; HDL-C, high-density lipoprotein cholesterol; uCa: 24- hour urine total calcium. FBG, fasting blood glucose; TC, total cholesterol; LDL-C, low-density lipoprotein cholesterol; The points (.) in the table represent variables with a coefficient of 0.

Note: According to the combination methods of multivariable stepwise Cox analysis and Cox-LASSO, age, gender, eGFR, urine ACR, albumin, hemoglobin, medical history of T2DM, and hypertension were considered in the final model. Between diastolic blood pressure and a history of hypertension, we chose the latter feature since it was readily available. Although 24-hour urine total calcium was statistically significant in the statistical analysis, it was less clinically actionable in practice and so was not included in the final model. While age was insignificant to the outcome, we still included it based on expert opinions.

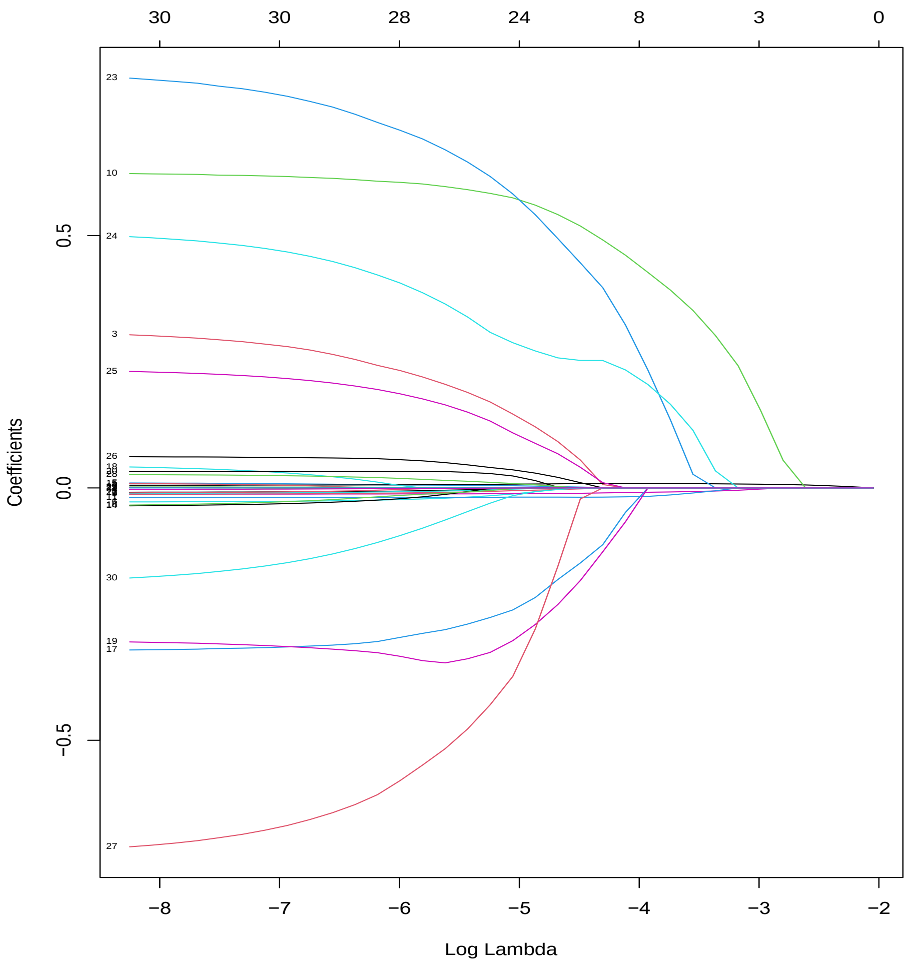


**Figure S2.** a. This Cox-LASSO figure shows that as the penalty coefficient lambda increases, the regression coefficient gradually compresses to 0 to reduce the features

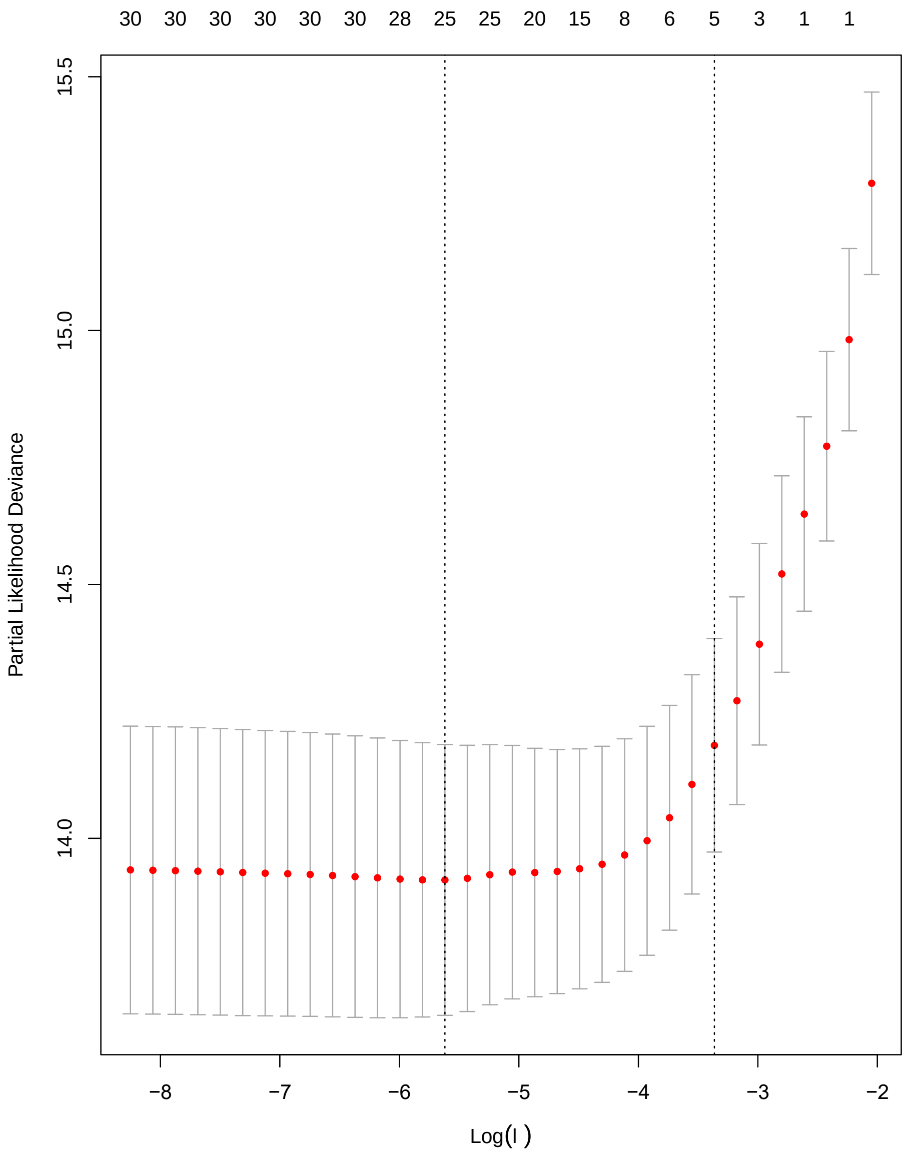


b. According to this Cox-LASSO cross-validation figure, as lambda increases, partial likelihood deviance gradually increases.

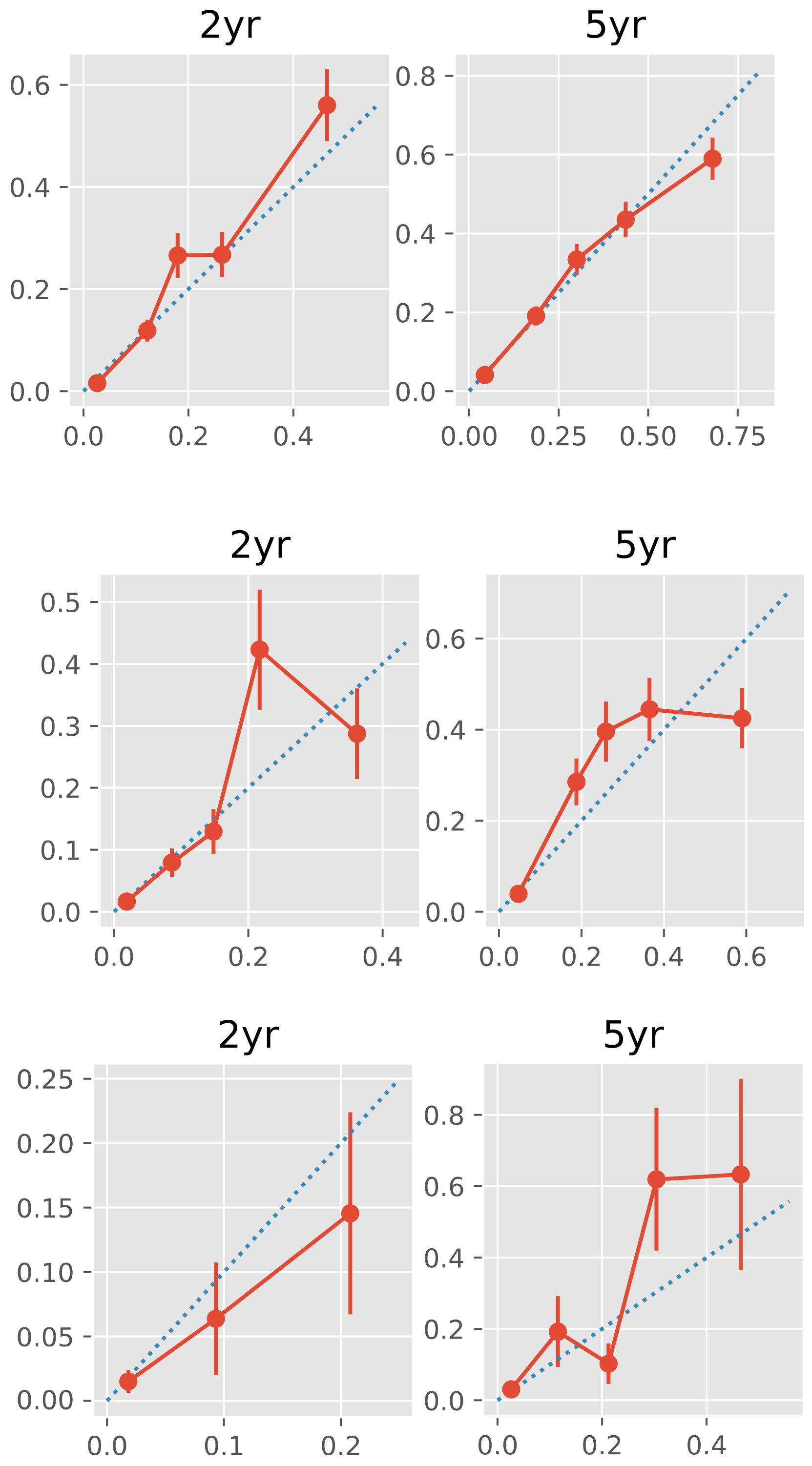


**Figure S3.** Visual plots of calibration for Coxph (2a.), SSVM(2b.), and XGBoost(2c.), 2 and 5- years in training, testing, and validation set.

**3a.** Calibration plots of actual outcome(y-axis) versus predicted risk(x-axis) for a **Cox** model in 2 (left) and 5 (right) years at training set(upper), testing set(middle) and validation set(down). The dashed line of identity helps for orientation: perfect predictions should be at the 45° line.

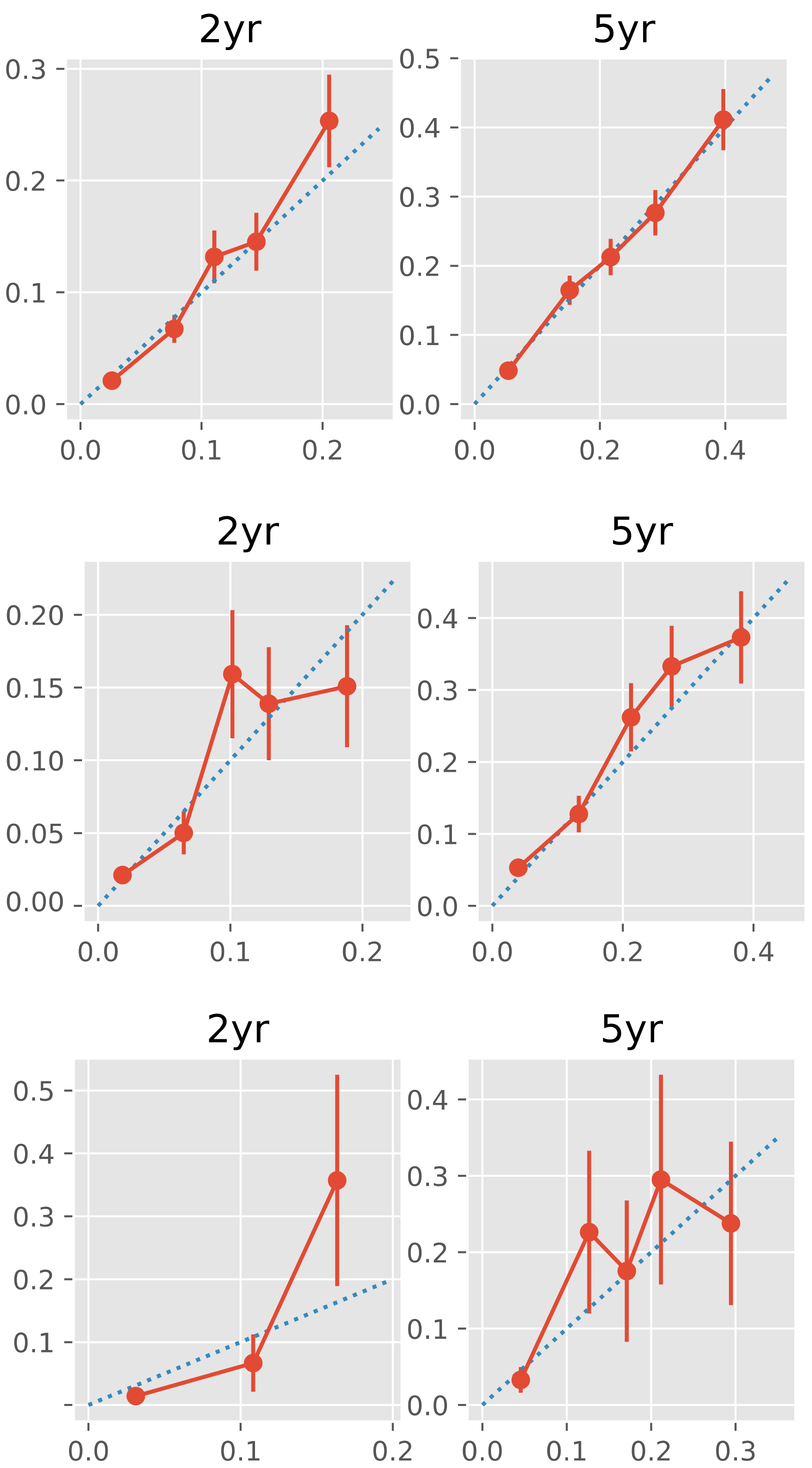


**3b.** Calibration plots of actual outcome(y-axis) versus predicted risk(x-axis) for a **SSVM** model in 2 (left) and 5 (right) years at training set(upper), testing set(middle) and validation set(down). The dashed line of identity helps for orientation: perfect predictions should be at the 45° line.

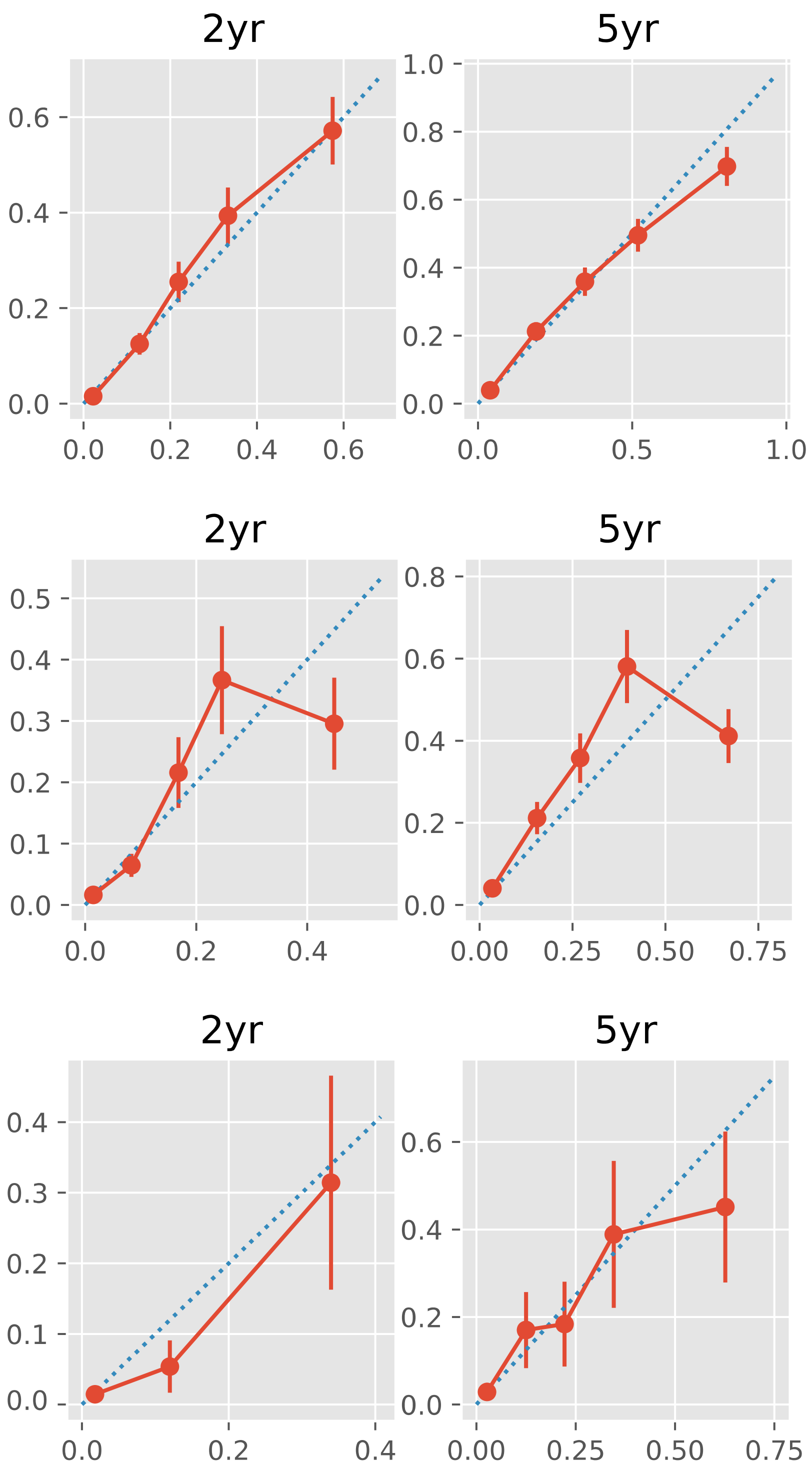


**3c.** Calibration plots of actual outcome(y-axis) versus predicted risk(x-axis) for a **XGBoost** model in 2 (left) and 5 (right) years at training set(upper), testing set(middle) and validation set(down). The dashed line of identity helps for orientation: perfect predictions should be at the 45° line.

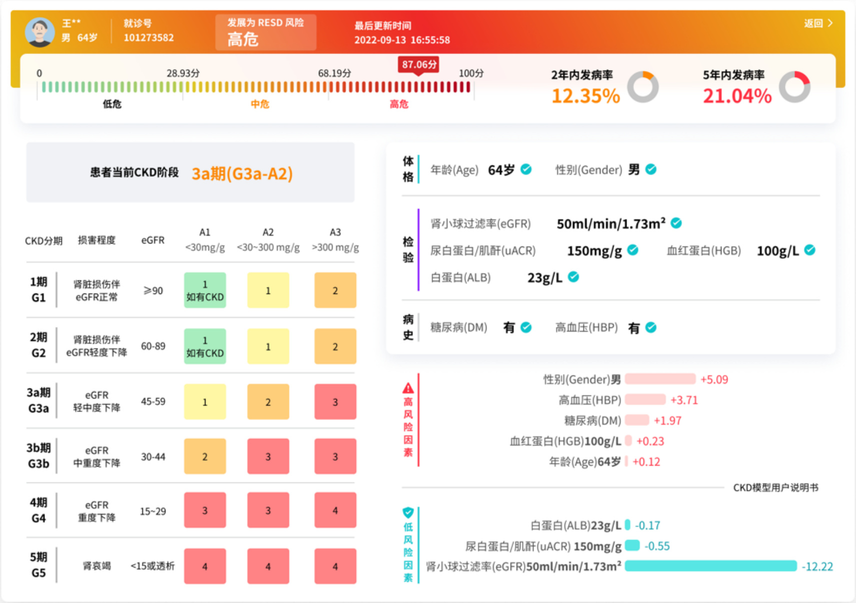


**Figure S4.** The PKU-CKD prognostic model clinical decision support system (Absolute risk predictions for KRT at 2- and 5-year for a scenario for a 64-year-old man with a history of diabetes and hypertension, an eGFR 50ml/min/1.73m$^2$ and a urine albumin-to-creatinine ratio of 150mg/g, an albumin 23g/L, and hemoglobin 100g/L).

Table S3. The equation to apply 2 or 5 years of the selected features to an Individual Patient.

| | Equations |
|---|---|
| Patient 5-year risk | 1 –S0(t) ^exp *f(x)*<br>1 – 0.9827 ^ exp (-0.0314 × (age) + 1.0268 ×male- 0.0449× (eGFR) + 0.6216 × (logACR) 0.2467-0.0459×ALB- 0.0184 ×HGB+ 0.4466×DM+ 1.3180 ×HBP) |

Where *f(x)*=linear predictor=$\beta_1 x_1 + \ldots + \beta_i x_i$

$\beta_{1\ldots}$ $\beta_i$ is represented the regression coefficient

$x_{1\ldots} x_i$ is represented the risk factor

S0(t) is the t-year survival rate for an individual with the average value of covariates in the risk equation ($x_{1\ldots} x_i$) and was 0.9969 at 2 years or 0.9827 at 5 years in the training set.

Table S4. Validation of 8-variable KFREs performance using CKD G3~G5 patients in 2 and 5 years.

8-variable equation, the patient's 2-year risk

| | *C*-index | Brier score |
|---|---|---|
| Original | 0.786 (0.013) | 0.08661916 |
| Regional calibrated original-non-north America | 0.786 (0.013) | 0.0849689 |

8-variable equation, the patient's 5-year risk

| | *C*-index | Brier score |
|---|---|---|
| Original | 0.75 (0.01) | 0.1599311 |
| Regional calibrated original-non-north America | 0.75 (0.01) | 0.1615296 |
